# Biological significance of sperm-independent calcium oscillations in immature oocytes of mice

**DOI:** 10.17912/micropub.biology.001748

**Published:** 2025-10-24

**Authors:** Sae Horiike, Woojin Kang, Minoru Ichinose, Ban Sato, Kenji Miyado, Hidehiko Ogawa

**Affiliations:** 1 Department of Bioscience, Graduate School of Life Science, Tokyo University of Agriculture, Setagaya-ku, Tokyo, Japan; 2 Laboratory Animal Resource Center, Transborder Medical Research Center, Institute of Medicine, University of Tsukuba, Tsukuba, Ibaraki, Japan; 3 Department of Reproductive Biology, National Research Institute for Child Health and Development, Setagaya-ku, Tokyo, Japan; 4 Department of Life Sciences, School of Agriculture, Meiji University, Tama-ku, Kawasaki, Kanagawa, Japan

## Abstract

Sperm-independent Ca ^2+^ oscillations are induced in immature oocytes, and presumably contribute to oocyte quality; however, its physiological role remains unclear. We studied the significance of Ca ^2+^ oscillations in ovarian functions using extramitochondrial citrate synthase ( *eCs* )-deficient (KO) female mice. In wild-type mice, the percentage of Ca ^2+^ oscillation-induced oocytes gradually decreased during their juvenile period, and dropped at the beginning of their adult period, whereas its percentage reduced more slowly in juvenile *eCs* -KO mice. Moreover, ovarian follicles containing two oocytes were frequently observed in ovaries of adult *eCs* -KO female mice. We assume that eCS suppresses spontaneous Ca ^2+^ oscillations, probably maintaining ovarian functions.

**Figure 1. Sperm-independent calcium oscillations in immature oocytes:**
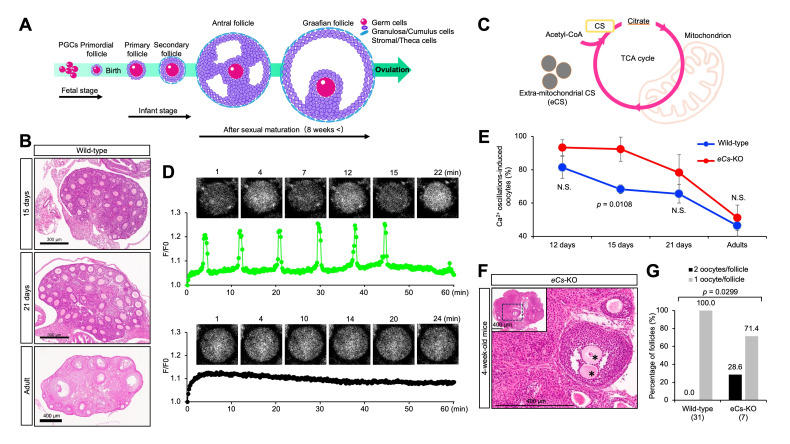
(A) Advances in oocyte maturation. (B) Changes in the number of ovarian follicles from juvenile to adult wild-type mice. Ovarian sections were stained with hematoxylin–eosin. (C) Cooperative function of two citrate synthases, CS and extra-mitochondrial CS (eCS). (D) Patterns of calcium (Ca ^2+^ ) oscillations in the immature oocytes from ovaries of 12-day-old mice. Upper graph, oocyte with Ca ^2+^ oscillations; lower graph, oocyte without Ca ^2+^ oscillations. (E) Percentage of Ca ^2+^ oscillations-induced oocytes. Ca ^2+^ oscillations were monitored in the immature oocytes isolated from ovaries of female mice aged at 12, 15, and 21 days, and 8-12 weeks old (adult). N.S.: not significant. Values are expressed as mean ± standard error of the mean. (F) An ovarian section of adult *eCs* -deficient ( *eCs* -KO) mice. Asterisks indicate two oocytes in a single follicle. The section was stained with HE. Fisher’s exact test was performed between 4-week-old *eCs* -KO and wild-type female mice. The parentheses indicate the number of mice examined.

## Description

Ovarian folliculogenesis (folliculogenesis) is the biological process by which ovarian follicles develop and mature (Sanchez and Smitz 2012) ( Figure 1A ). This leads to the release of mature oocytes during ovulation and the production of hormones including estrogen and progesterone. Folliculogenesis consists of the following key stages: (1) primordial follicle formation, (2) follicle activation, (3) transition from primary to secondary follicles, (4) antral follicle (tertiary follicle) formation, and (5) preovulatory (Graafian) follicle formation, which is a highly regulated cyclic process that ensures the monthly development of mature follicles ready for ovulation in humans. Therefore, the disturbances in this process lead to pathological symptoms such as polycystic ovarian syndrome, premature ovarian insufficiency, and infertility (Pandey et al. 2025).

Mouse follicles grow faster, have fewer granulosa layers, and smaller oocytes, while human follicles are larger, more complex, and supported by longer-lasting hormonal cycles. Although there are various differences between human and mouse folliculogenesis, there are also commonalities. During folliculogenesis, the number of immature oocytes in the ovaries decreases naturally from birth both in humans and mice ( Figure 1B ).

Repeated rises in cytoplasmic calcium levels, known as Ca²⁺ oscillations, regulate numerous biological processes. During fertilization, the sperm-derived factor phospholipase Cζ1 (PLCζ1) initiates these oscillations, leading to the resumption of cell division in oocytes (Swann 2025). Citrate synthase (CS) is localized in the mitochondrial matrix where it catalyzes the reaction between acetyl-coenzyme A (acetyl-CoA) and oxaloacetate to form citric acid ( Figure 1C ). In mice, the extramitochondrial form of citrate synthase (eCS) is encoded by a distinct gene, while in humans, it is produced through alternative splicing of the *CS* gene. eCS acts as a secondary factor that initiates Ca²⁺ oscillations during fertilization, transmitting signals from the sperm to the oocyte (Kang et al. 2020). Specifically, eCS induces an initial Ca²⁺ spike through a mechanism that does not involve PLCζ1. Additionally, sperm-independent Ca²⁺ oscillations occur in immature oocytes isolated from the ovaries of both juvenile and adult female mice. The presence of Ca ^2+^ oscillations may contribute to oocyte quality; however, their physiological role and molecular mechanisms remain unclear.

We studied the significance of Ca ^2+^ oscillations in the ovarian functions using *eCs* -deficient (KO) female mice. When Ca ^2+^ oscillations were monitored, oocytes with or without Ca ^2+^ oscillations were observed ( Figure 1D ). The percentage of immature oocytes from the ovaries of juvenile mice gradually decreased, and the majority of oocytes were not induced in adult mice ( Figure 1E ). This tendency was similar between *eCs* -KO and wild-type mice; however, the percentage of Ca ^2+^ oscillation-induced oocytes decreased slightly in *eCs* -KO mice compared to that in wild-type mice. In oocyte from 15-day-old mice, there was significant difference between *eCs* -KO and wild-type mice (92.3 ± 2.6% vs. 68.3 ± 7.2%; *p* = 0.0108). Moreover, ovarian follicles containing two oocytes were frequently observed in the ovaries of 4-week-old *eCs* -KO female mice ( Figure 1F ), and statistically increased in *eCs* -KO mice, compared with wild-type mice ( *p* < 0.0299) ( Figure 1G ). From this result, we have assumed that contrary to sperm-carried eCS, eCS could suppress spontaneous Ca ^2+^ oscillations in the ovaries, presumably maintaining some ovarian functions.

The biological significance of Ca²⁺ oscillations in immature oocytes lies primarily in their preparation for meiotic progression and developmental competence. While mature oocytes display Ca²⁺ oscillations during fertilization, immature oocytes also exhibit Ca ^2+^ oscillations (Swann 2025).

Anaerobic organisms like eubacteria and archaea utilize the reverse TCA (rTCA) cycle to synthesize carbon-based compounds from carbon dioxide and water (Wachtershauser 1990). The rTCA cycle can proceed in both directions, either enzymatically or non-enzymatically, to drive reduction reactions involving carbon compounds, depending on environmental conditions (Keller et al. 2017; Muchowska et al. 2017). Most enzymes associated with the TCA cycle also participate in the rTCA cycle. In particular, three key enzymes—ATP-citrate lyase, 2-oxoglutarate synthase, and fumarate reductase—are known to play crucial roles in its function (Tang and Blankenship 2010). Remarkably, CS regulates the reductive and oxidative directions in the rTCA cycle of *Thermosulfidibacter takaii* ABI70S6T, suggesting that this reversible enzyme converts citrate to acetyl-CoA (Nunoura et al. 2018).

The "two-waves of oogenesis" model in mammals describes the development of ovarian follicles in distinct waves during fetal and early postnatal life (Kissel et al. 2000; Dai et al. 2022). This concept contrasts with the long-held dogma that females are born with a fixed, non-renewable supply of oocytes. The two-wave model is a modern area of study, particularly in mice, and remains a subject of intense scientific debate in the context of postnatal oogenesis in humans. In oocyte from 15-day-old mice, there was significant difference between *eCs* -KO and wild-type mice ( Figure 1E ), implying that two types of oocytes would be mixed in the ovaries of 15-day-old *eCs* -KO mice.

Citrate functions as a suppressor of harmful events, such as oxidative stress and inflammation, and helps protect tissues from damage. While it has been shown to regulate various biological processes, its full range of roles remains poorly understood. Our results contribute to our understanding of citrate and Ca ^2+^ oscillations in ovarian functions.

&nbsp;

## Methods

**Animals**

Mutant mice were generated from C57BL/6-derived embryonic stem cell clones by injection into blastocysts from C57BL/6 mice with a genetically deleted *Csl* ( *eCs* ) (Csl ^tm1(KOMP)Vlcg^ ; ID14519). These mice obtained from the Knockout Mouse Project (KOMP) repository (an NCRR-NIH-supported strain suppository; www.komp.org ) (Kang et al. 2020). Homozygous mice (C57BL/6 genetic background) were generated by the subsequent intercrossing of heterozygous animals. As control, 8-12-week-old male C57BL/6J mice were purchased from Japan SLC Inc. (Shizuoka, Japan). All the mice were housed under specific pathogen-free conditions. Food and water were provided ad libitum. All the animal experiments were approved by The Institutional Animal Care and Use Committee of the National Research Institute for Child Health and Development (Experimental number, A2004-004).

**Monitoring of Ca ^2+^ oscillations in immature oocytes**

To monitor changes in intracellular Ca ^2+^ concentration, immature oocytes were collected from the ovaries of mice aged 12, 15, and 21 days, and 8-12 weeks old (adult). Immature oocytes were collected from WT and eCs-KO female mice. The number of female mice used was 3, 4 or 5 in each stage and genotype. In order to monitor Ca ^2+^ oscillations, 50-100 oocytes were placed in a single drop of fertilization medium (Toyoda-Yokoyama-Hoshi medium; TYH medium) and incubated in TYH medium containing the Ca ^2+^ -sensitive fluorescent dye, Oregon green 488 BAPTA-1 AM (final concentration of 2 μM, Molecular Probes, Invitrogen, Carlsbad, CA) for 15 min at 37 °C in a CO _2_ incubator. The cells were then washed three times with TYH medium (5 min each). Fluorescent images of the oocytes were captured using a highly sensitive CCD camera (Andor Technology, Belfast, UK), using software to operate the camera (Yokogawa, Tokyo, Japan) every 10 s. Fluorescence intensity was measured using Andor IQ imaging software (Andor Technology). Each fluorescent image (F) was subtracted from the image before injection or from the image with the lowest fluorescence intensity (F0). The fluorescence intensity of individual oocytes was measured within a user-selected region covering most of the oocyte area. The mean intensity over the same area for each image in the time series was automatically analyzed. Changes in fluorescence intensity were reported as the F/F0 ratio, as previously described (Kang et al. 2020).

**Statistical analysis**

Comparisons were made using one-way analysis of variance following Scheffe’s method, Mann–Whitney *U* -test, or Fisher’s exact test. The statistical significance was defined as *p* < 0.05. Results are expressed as the mean ± standard error of the mean.
